# Post-translational modifications in liquid-liquid phase separation: a comprehensive review

**DOI:** 10.1186/s43556-022-00075-2

**Published:** 2022-05-11

**Authors:** Jingxian Li, Mengdi Zhang, Weirui Ma, Bing Yang, Huasong Lu, Fangfang Zhou, Long Zhang

**Affiliations:** 1 MOE Laboratory of Biosystems Homeostasis & Protection and Innovation Center for Cell Signaling Network, Life Sciences Institute, Zhejiang University, Hangzhou, 310058 China; 2 School of Medicine, Zhejiang University City College, Hangzhou, 310015 Zhejiang China; 3 Institutes of Biology and Medical Science, Soochow University, Suzhou, 215123 P. R. China

**Keywords:** Liquid-liquid phase separation, Post-translational modifications, Poly (ADP-ribosyl)ation, Neurodegenerative diseases

## Abstract

Liquid-liquid phase separation (LLPS) has received significant attention in recent biological studies. It refers to a phenomenon that biomolecule exceeds the solubility, condensates and separates itself from solution in liquid like droplets formation. Our understanding of it has also changed from memebraneless organelles to compartmentalization, muti-functional crucibles, and reaction regulators. Although this phenomenon has been employed for a variety of biological processes, recent studies mainly focus on its physiological significance, and the comprehensive research of the underlying physical mechanism is limited. The characteristics of side chains of amino acids and the interaction tendency of proteins function importantly in regulating LLPS thus should be pay more attention on. In addition, the importance of post-translational modifications (PTMs) has been underestimated, despite their abundance and crucial functions in maintaining the electrostatic balance. In this review, we first introduce the driving forces and protein secondary structures involved in LLPS and their different physical functions in cell life processes. Subsequently, we summarize the existing reports on PTM regulation related to LLPS and analyze the underlying basic principles, hoping to find some common relations between LLPS and PTM. Finally, we speculate several unreported PTMs that may have a significant impact on phase separation basing on the findings.

## Introduction

Rapid technological development has facilitated the understanding of biology at the molecular level, which provides a new perspective to known life processes. For example, in 1899, Edmund Beecher Wilsonin proposed that the cytoplasm consists of small, suspended droplets [1]. The basic biological principle of LLPS is simple; when macromolecules are subjected to external stimuli or changes, they form condensed liquids with small volumes and high concentrations by gathering together in a limited space. Accordingly, cells can regulate physical processes at the protein level to avoid time- and energy-consuming translation processes such that existing proteins are effectively used to respond in time and achieve intracellular functional compartmentalization without additional energy inputs [1, 2]. Many kinds of proteins can form liquid-like droplets. Recently, stimulator interferon genes (STINGs) were found to undergo liquid-liquid phase separation (LLPS) when overactivated by virus stimulation and form a sponge-like three-dimensional structure to adsorb excessive tank bind kinase 1 and insulate interferon regulatory factor 3 to avoid inflammatory responses [2]. Nevertheless, the driving forces of STINGs droplets formation at the micron level still remain unknown [3–5]. Consequently, further studies are needed to gain deep insights in to the mechanism of LLPS.

**Fig. 1:**
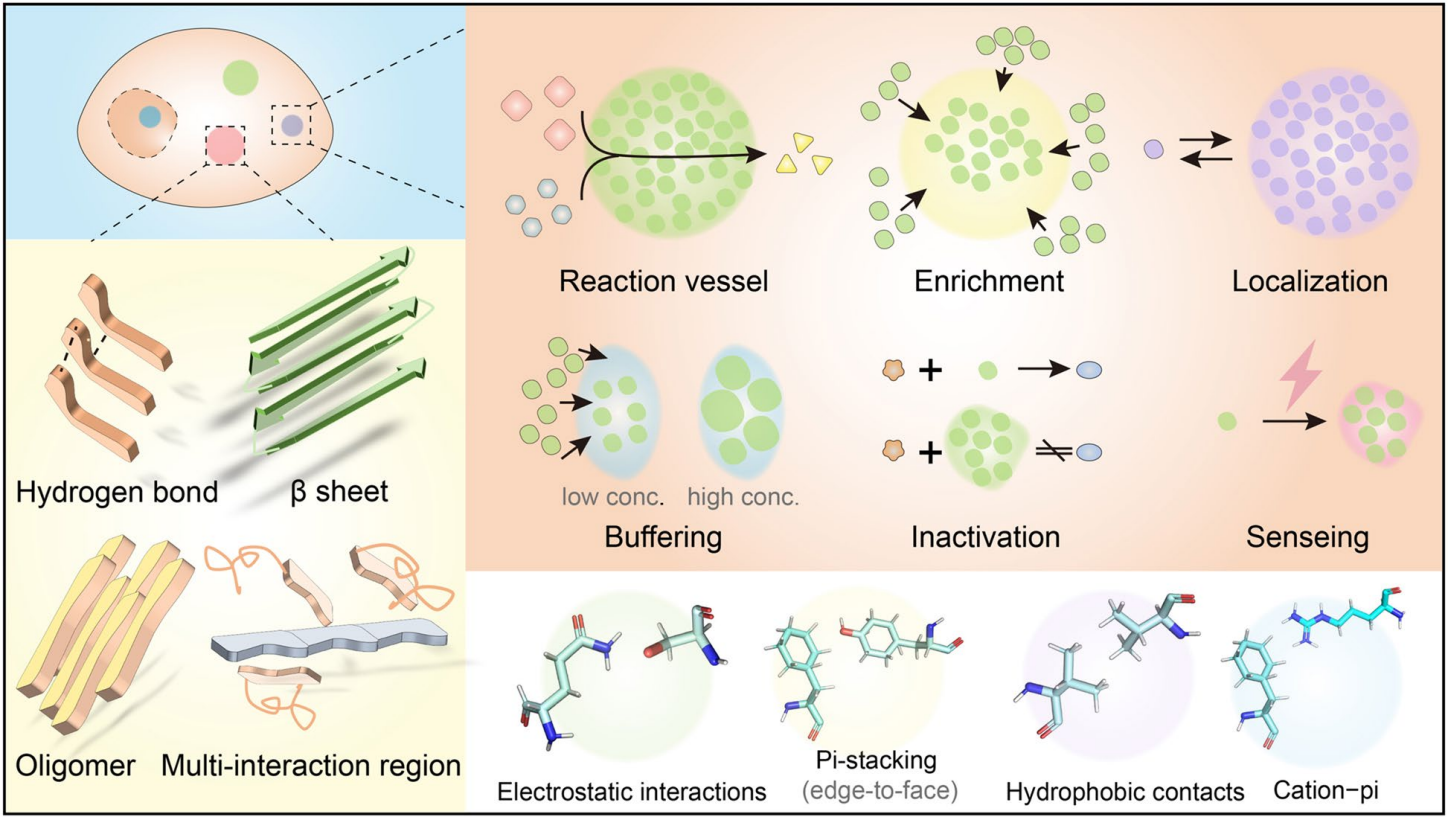
Function of LLPS and forces that drive it. LLPS has been found to be involved in a variety of physiological processes, which can be divided according to their functions, including reaction vessel, enrichment, localization, buffering, inactivation, and sensing. Weak, transient, and short-ranged interactions, including electrostatic interactions, π-stacking, hydrophobic contacts, and cation-π interactions, are the basic driving forces of LLPS. Covalent or strong intermolecular interactions and secondary structures, including the hydrogen bond, β-sheet, oligomer, and multi-interaction region, also contribute to LLPS

Recently, phase separation has been detected in multiple biological processes, such as DNA damage repair, mRNA transcription, protein translation, membrane receptor assembly, cytoskeleton stabilization, embryonic development, and plant floral cycle regulation [3–9]. LLPS is involved in different physiological processes, and its basic functions include providing reaction vessel, enrichment, localization, buffering, inactivation, and sensing (Fig. 1) [10–12]. For instance, in mRNA transcription, elongated nucleic acids provide ideal platforms for the interaction of multi proteins, which accounts for many transcription factors in LLPS-dependent processes [13]. Another example, P-body participates in the renewal of mRNA by concentrating enzymes in the cytoplasm, while shielding numerous mRNA strands that do not require translation from the protein [14].

There is a positive correlation between compartmentalization and protein post-translational modification (PTM) in evolution [15, 16]. PTMs, similar to LLPS, a fast-regulating and reversible processes that govern life procedure, are the subsequent steps of protein translation, which includes chemical groups attached to or divested from the substrates. As for compartmentalization, specific compartments allow physiological processes to function independently and simultaneously, which makes cells respond to external stimuli with a low energy expenditure. The compartmentalization of the nucleus insulates transcription and translation processes, making PTM a common strategy in the eukaryotic cell [17]. Based on this principle, it is considered that the LLPS, which represents a form of compartmentalization, is related to PTM.

A comprehensive analysis of the aforementioned phenomena and biological functions will allow the researchers to gain in-depth understanding about the relationship between LLPS and PTM.

## The driving force and theory of LLPS

A brief description of the LLPS process is necessary for a deep understanding of the role of PTMs in phase separation. In physics, phase separation occurs either when a molecule reaches the upper limit of dissolution or when the entropy of the solution remains at the maximum and repels the molecule from the solution system [18, 19]. A detailed explanation on the law of protein solubilization in solution can be found from the entropy perspective [20], in which the balanced solution tends to hold more protein particles to maintain a large entropy value in a stable case [21]. The aggregation of biological macromolecules causes the number of particles in the solution to decrease below the equilibrium curve, and unless there is an additional energy input, the solution will be more inclined to reject them to the solution system to obtain a higher entropy value, thereby separating into interactive small volume high concentration droplets and a large volume low concentration medium [22]. It worth noting that “aggregation” here means the reversible assemblies of biomacromolecules.

The driving force of phase separation is considered to be the weak interactions between amino acid groups and other macromolecules, including homologous proteins, non-homologous proteins, or nucleic acids. The weak interactions include π-stacking, electrostatic, cation-π, and hydrophobic contacts [6, 9, 23, 24]. Therefore, the specific types of amino acid side chains, such as non-polar amino acids, charged amino acids, and aromatic amino acids, can promote phase separation [25, 26]. In the study of protein structure, the disordered regions with the variable and rapid interactions are rich in these amino acids, and many different conformations exist simultaneously. In previous studies, the short distance, transient, and weak interactions between amino acid side chains in intrinsically disordered regions (IDRs) was considered to be the driving force for phase separation [27].Notably, IDRs are only the primary structure and do not have secondary structure regions contributing to protein folding and aggregation [28, 29]. However, the interaction between macromolecules and the folding structure can regulate the phase separation by adjusting the distance and quantity relationship between macromolecules. The existing prediction programs for IDRs tend to ignore the isolated amino site, which might be important in driving phase separation, because of the use of the accumulation calculation method [30]. This might be the reason why some reported proteins can still undergo phase separation after the disordered region is truncated [31–33]. For example, in the SARS-CoV2019 nucleic protein NP studies, mitochondrial antiviral signaling protein, which is a crucial scaffold protein in the antiviral signaling pathway, was found to retain its ability to undergo LLPS after the deletion of IDR regions [32].

**Fig. 2:**
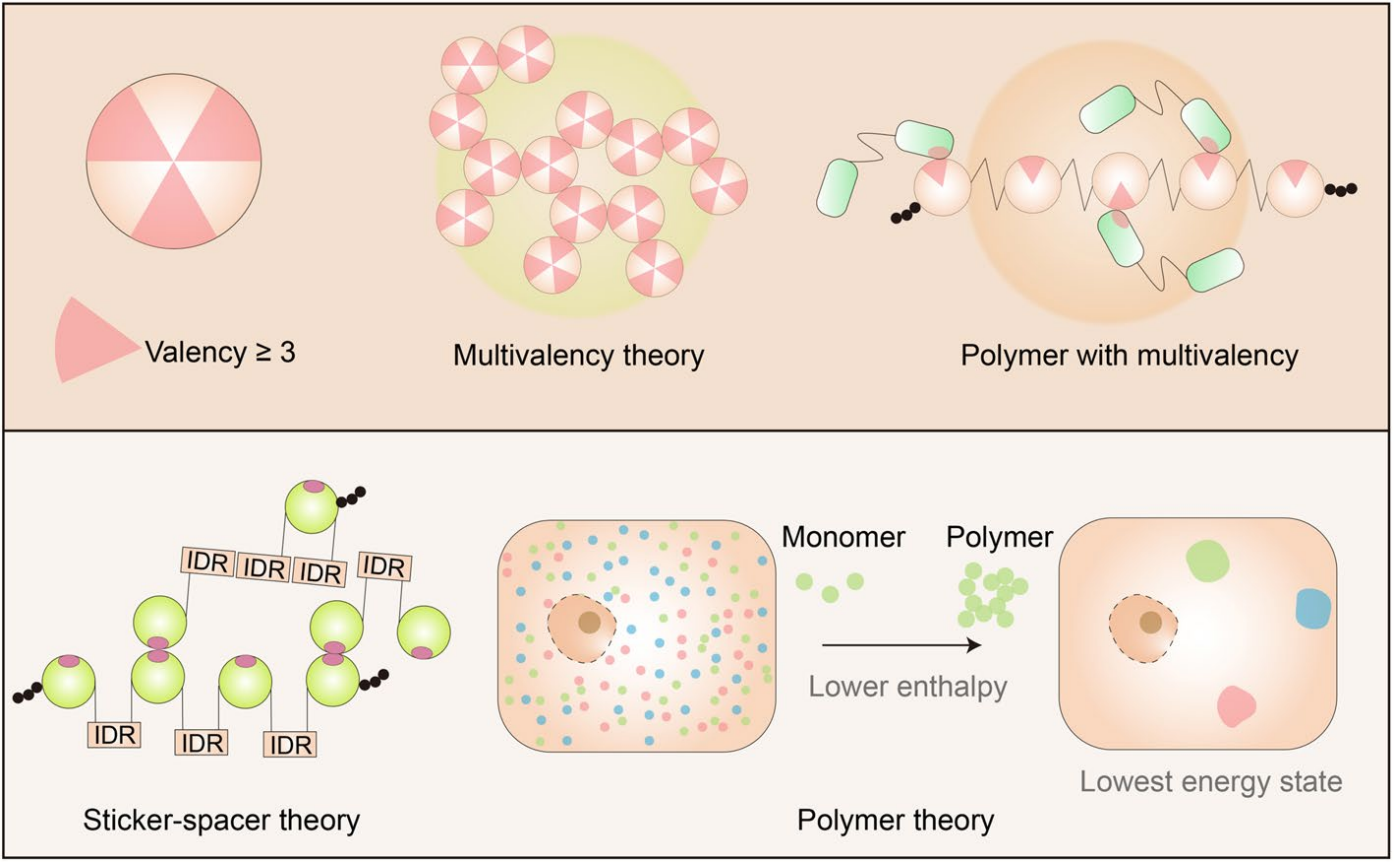
Theories on phase separation. Three different theories were proposed to explain LLPS. Multivalency theory indicates protein(s) or a peptide or a part of nucleotides has more than three valences to bind with other molecules, which can interact with others in more than two dimensions. Polymers and long-chain structures are ideal for the multivalency theory. Sticker-spacer theory is similar to the multivalency theory, except it focuses more on the ordered and interaction regions. Polymer theory is based on the hypothesis that the attraction between LLPS proteins is much stronger than its attraction with its surrounding environment. Therefore, the LLPS system can reach the lowest energy state because of the low enthalpy caused by polymerization

Thus far, three different theories have been proposed to explain LLPS, namely, the multivalency theory, sticker-spacer theory, and polymer theory (Fig. 2) [20, 23, 34, 35]. The multivalency theory and the polymer theory provide different summaries of entropy and aggregation reasons [23, 36–38]. The total loss of - primarily translational - entropy upon phase separation is less if the interacting modules are chained together into a linear multivalent protein. For example, the LLPS of the proline rich motif (PRM4) and Src homology 3 (SH3) are strongly affected by the number of repeats and exhibit a positive correlation [23]. Notably, the multivalency theory is also used to explain the decrease in the liquid-gel transition threshold. Correspondingly, the sticker-spacer theory complements the multivalency theory, with more focus on the “order region” [34]. The polymer theory is used in explaining the gathering of biomolecules that driven by distinct interaction tendencies, which overcomes the decrease of entropy after polymerization [39].

Phase separation is a highly dynamic process [40]. The original dispersed protein must undergo certain changes to achieve “aggregation” when cells release recruitment signals in a specific range and time. After the function is performed, the protein accepts a new signal and returns into solution without changing the existing intracellular protein expression level. Rapid protein PTM can be an ideal way to regulate phase separation [35, 41]. To systematically understand the relationship between post-translational protein modifications and phase separation, we summarized reports on protein phosphorylation and acetylation in phase separation and devote more attention to changes in physical properties caused by modifications in regulating phase separation. Based on these results, we predicted several previously unreported PTMs that may be involved in the regulation of phase separation (Table 1).

**Table 1:** Multivalency in phase separation

Year of publication	Driving force or crucial amino acids	PTMs	Reference
2016	Covalent and non-covalent interaction between PRM and SH3	Phos.	[23]
2016	Electrostatic interactions		[42]
2016	(multi-valent) cation and (hydrophilic) protein		[43]
2016	Hydrophobic interaction		[44]
2017	SH3 proline-rich motif-induced hydrophobic interaction		[45]
2017	Forces between aromatic amino acids, especially, tyrosine	Phos.	[46]
2018	Asparagine, phenylalanine, and tyrosine are important to phase separation		[47]
2018	Proline-rich motif		[48]
2018	Multivalent hydrophobic and polar interaction	Ubiquitin binding	[49]
2018	Electrostatic interactions		[24]
2019	Phosphorylation and proline-rich motifs	Phos.	[50]
2019	Hydrogen bonding, π/sp2, and hydrophobic interactions		[27]
2019	Phosphorylated threonine residues	Phos.	[51]
2020	Multivalent electrostatic interactions		[52]
2020	Multivalent covalent interactions between long K48Ub chains and UBA domains		[53]
2020	Multivalent contacts through arginine’s guanidinium ion		[54]
2020	Five β-strands and π–π stacking		[55]
2020	Multivalent electrostatic interactions and hydrophobic interactions		[56]
2020	Tyrosine	Phos.	[57]
2020	Intermolecular electrostatic interactions		[58]
2020	Nonspecific hydrophobic interactions as well as hydrogen bonds, salt bridges, and π–π and cation−π interactions		[59]
2021	Phosphorylation-induced cluster	Phos.	[60]
2021	Phosphorylation-induced interaction	Phos.	[61]
2021	Multiple SPOP-binding (SB) motifs		[62]
2021	DD domain interaction	Ace.	[32]

## Effect of physical conditions on LLPS

**Fig. 3:**
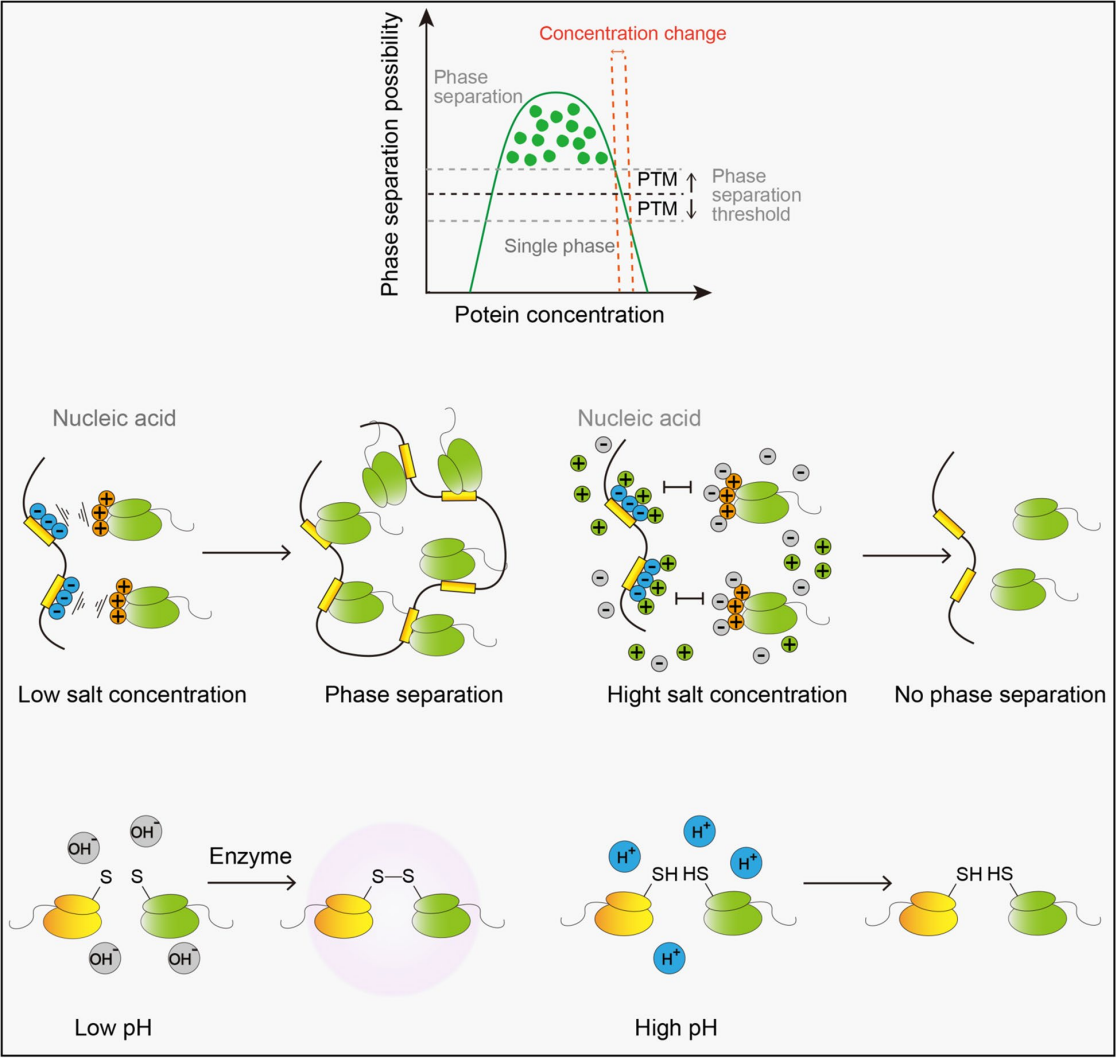
Environment stimulus in regulating LLPS. Phase separation can only occur in a relatively narrow region under ideal conditions, and PTMs may regulate LLPS by changing the thresholds of substrates. Salt ions in the environment can bind to the originally charged substrate via electrostatic interaction and inhibit LLPS by neutralizing electrostatic interaction. Similarly, the redox activity of cysteine is regulated by pH and therefore controls the formation of disulfide

Protein solubility is affected by the physical properties of the solution, particle size, and aggregation status of the biomolecules. The internal environment of the cell is generally maintained in a stable state, and a significant transformation of its physical properties is often a manifestation of the cell stress. Cells benefit from this property while responding to external stimuli [63, 64]. There are different properties that influence LLPS (Fig. 3), including temperature, pH, and ion changes as well as the changes in reactive oxygen species (ROS) and hydrogen bonds. It is conventional to test the LLPS of purified proteins in a series of temperature gradients. However, under normal circumstances, the temperature of the human body is relatively constant, and only tissues in direct contact with the environment are likely to experience temperature changes. In addition, the ways in which purified proteins are subjected to temperature regulation is not similar. For example, the LLPS between poly(A)-binding protein and RNA-recognition motifs is enhanced at low temperatures owing to weak forces between them [65]. But early flowering 3, which is a constitutively expressed protein that regulates plant rhythm, tends to undergo LLPS at high temperatures because of the breakdown of protein-solution binding [9]. In addition, hydrophobic effect can also explain this behavior [66–68]. The second factor that influences LLPS is pH and ion changes [69–71]. The concentration of H^+^ mainly affects the ion-bond-induced LLPS by neutralizing the original positive and negative charges [72]. For instance, damage to the endoplasmic omental system owing to viral infestation leads to the outflow of zinc ions into the cytoplasm, promoting the phase separation of cyclic GMP-AMP synthetase [73]. This generally occurs in small areas at a micron level and is comparable to other categories of separation. Another factor is the changes in ROS and hydrogen bonds [9, 27]. In the regulation of the plant flowering cycle, ROS was found to be associated with the LLPS for terminating flowers by regulating the formation of disulfide bonds. Another factor that is easy to overlook is the size of the molecule [21, 22, 74]. Aggregated molecules can lead to phase separation by lowering the entropy of the solution. The aggregation of biological macromolecules also strengthens the weak interaction between molecules [21]. When forming a polymer, amino acids, which were originally spatially far apart, are enriched and stacked in a small area [75]. For example, positively charged amino enrichment strongly attracts negatively charged groups, whereas the stacking of benzene rings promotes the formation of π bonds [76–78]. It accounts for the occurrence of many diseases in the nervous system. For instance, α-synuclein (α-Syn) is a key protein in amyloid formation, which is directly linked to Parkinson’s disease pathogenesis [79]. The α-syn stack provides a platform for enhancing the interactions of the N-terminus and hydrophobic non-amyloid-β component domain [80].

## The regulation of various PTMs on LLPS

Interactions are considered to be the main reason for LLPS regulation; covalent bonds, non-covalent bonds, electrostatic interactions, hydrophobic forces, and hydrophilic forces are reported to be affected by PTM. Therefore, the LLPS of proteins could be regulated by various PTMs, including phosphorylation, acetylation, poly (ADP-ribosyl)ation (PAR), ubiquitination and methylation. Early studies on LLPS mainly focused on the relationship between phase separation and phosphorylation modification which is widely reported in many proteins involved in different physical processes [81]. For instance, The protein fused in sarcoma (FUS), which is a well-researched protein in the field of LLPS, is phosphorylated at several sites, including pS26, pS42, pS61, pS84, and pS131 [82]. These modifications regulate the interaction between FUS and DNA, and the N-terminal serine/tyrosine-rich region is also considered to be a domain that influences LLPS. However, the basic principles vary from study to study [23, 46, 50, 51], and they are summarized below.

### Two sides of phosphorylation regulation of LLPS

**Fig. 4:**
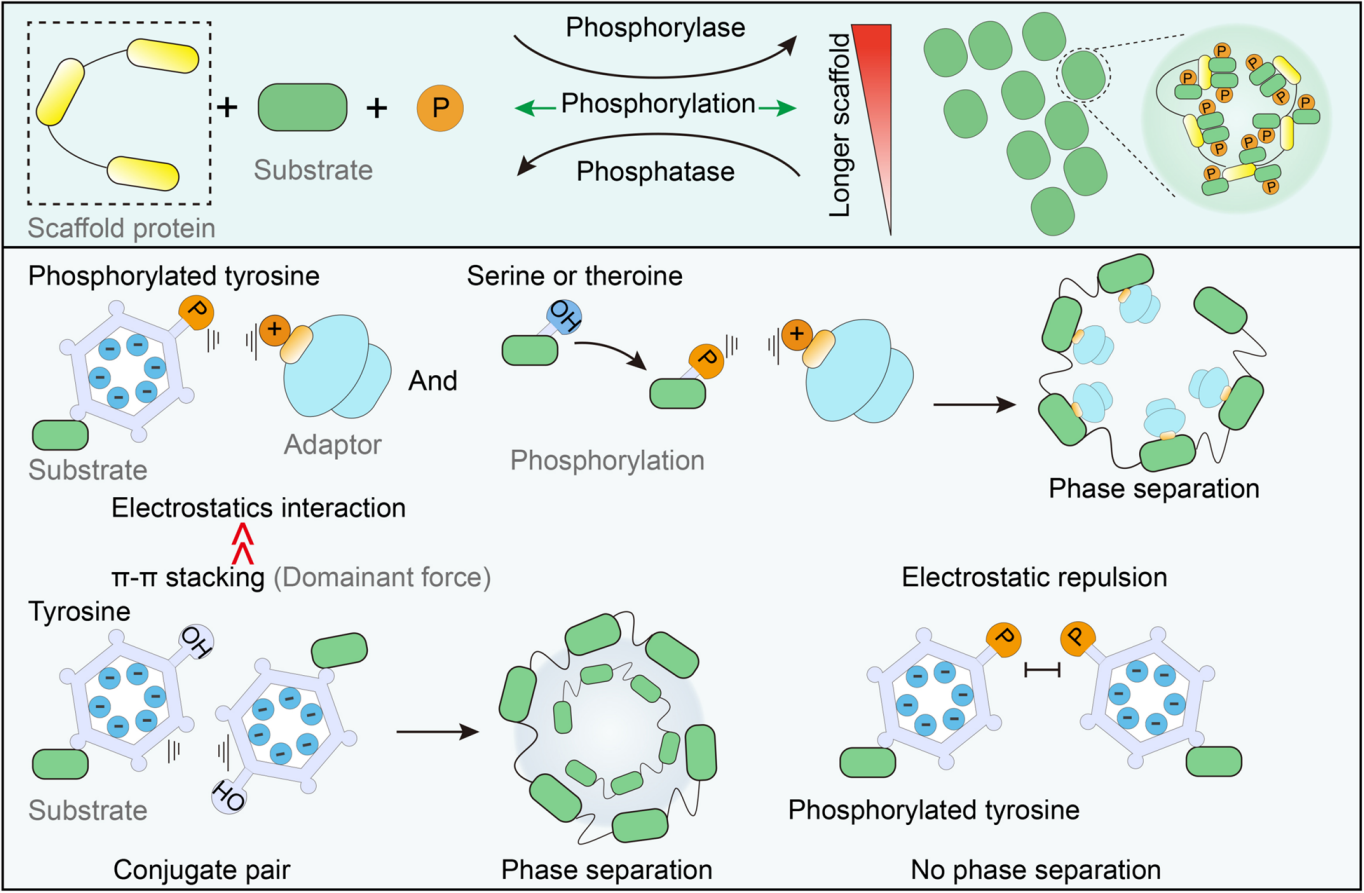
Phosphorylation in LLPS. Phosphorylation and LLPS have similarities such that they are both reversible and rapid in response to stimuli. Electrostatic interactions of serine, threonine, and tyrosine increases after phosphorylation, but the pi-stacking force is severely damaged in phosphotyrosine. Considering that pi-stacking is more dominant in controlling LLPS, phosphorylation on tyrosine is commonly inhibitory

Phosphorylation is an ideal method for the regulation of protein separation. Phosphorylation can occur rapidly and reversibly in various subcellular organelles, enabling an easy response to sudden stimulation changes throughout the cell. The regulation of phosphorylation is bidirectional [41] (Fig. 4) such that the addition of phosphate groups to uncharged amino acids results in negatively charged amino acids, which can enhance both electrostatic attraction and electrostatic repulsion. Therefore, phosphorylation can either enhance or inhibit phase separation. Interactions between proteins and nucleic acids are sensitive to charge changes. In the study of the Histone H1 (H1) phase separation, the phosphorylation modifications of three serines in the carboxyl domain of H1 protein inhibited their phase separation, and a 10% change in the surface charge of the protein in this process was sufficient to significantly affect the binding capacity of the protein to DNA as well as the cohesion of DNA and protein [83]. An earlier basic study on the role of phosphate peptide segments in phase separation revealed that phosphorylation at even one site can affect the electrostatic interaction between peptides and the negatively charged RNA and thus affect phase separation [46]. Evolutionary perspectives also support the idea that cells specifically regulate phase separation with phosphorylation modification. The conservative analysis of FUS protein sequences indicated that the phosphorylation sites associated with phase separation in mammalian FUS were selectively preserved during evolution [84]. However, there are often multiple phosphorylation sites on a single protein, which may have opposite functions. When the kinase content is abundant, non-specific phosphorylation modification inevitably affects phase separation. The study of fragile-X mental retardation protein (FMRP) and calcium-activated neutral proteinase 1 (CARPN1) phase separation demonstrates the use of phosphorylation modification by cells to regulate the separation of complexes from RNA. The phosphorylation of FMRP on serine, threonine, and tyrosine on CAPRIN1 affects the binding of SC1RNA, and de-adenylation activity owing to the phase separation facilitates cascade regulation [85].

**Fig. 5:**
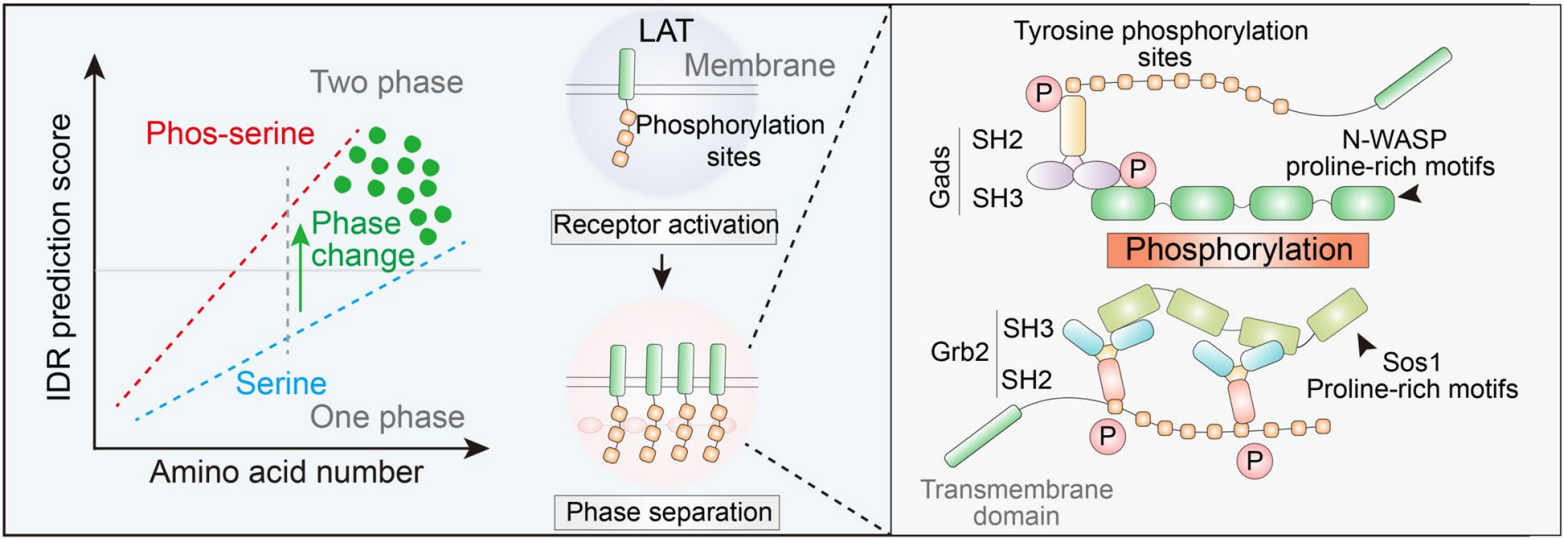
Phosphorylation in LAT LLPS. Both phosphoserine and serine are basic parameters in IDR calculation. Activation and LLPS of LAT are related to phosphorylation-induced interactions among LAT, Gads, Grb2, and Sos1

We have summarized reports on the separation in the control of phosphorylation modification and identified three significant points. First, the number of reports on the phosphorylation of tyrosine is disproportionately large [81]. No more than 1% of phosphorylation occurs on tyrosine in general; however, considering the reports on phase separation, nearly 40% of them are related to tyrosine. For example, the study focused on protein phase separation related to tyrosine phosphorylation and complex assembly on the membrane surface. KRAS and DHPE form condensates on lipid membranes independently; however, only in the presence of the activation of T cells (LAT)/growth-factor-receptor-bound protein 2 (Grb2)/sevenless (SOS) can they undergo phase separation [86]. Notably, tyrosine phosphorylation influenced by temperature on the LAT also combines with Grb2/SOS to achieve phase separation (Fig. 5).

Second, most published studies conclude that phosphorylation on tyrosine inhibits phase separation; for instance, In the study of neurodegenerative diseases, Tau phosphorylation on T231 and S235 inhibits tubulin phase separation and the formation of microtubule [87]. The authors suggest that it is a common mechanism in condensate-mediated cytoskeletal assembly. In another example, Fyn, a tyrosine kinase, can phosphorylate the granule protein heterogeneous nuclear ribonucleoprotein A2 (hnRNPA2) on its low complexity domain and inhibit its aggregation and phase separation. The authors also discovered hnRNA2’s antagonizing function on the disease-related mutant D290V in *C. elegans* neurodegeneration [88]. It is reasonable to attribute this phenomenon to the phosphate inhibitory effect on the hydroxyphenyl group of the tyrosine side chain, which is more likely to interact with surrounding amino acids through electrostatic interactions with the positive charge, phenyl ring π bond stacking, and cationic π bond interaction [76, 78]. As for the explanation of negative regulation, tyrosine only relies on the electrostatic interaction of hydroxyl groups originally, but the addition of phosphate groups can significantly change these properties, by switching the original positive charge and blocking the stacking of the molecules. In addition, considering that the benzene ring plays a decisive role in LLPS, the effect of phosphorylation may predominately show its function by affecting π-bond stacking. Studies on FUS lead to the same conclusion. The tyrosine residues across FUS contribute equally to LLPS with regard to their position and IDR regions, and although the interaction between PRM and SH3 is not influenced by FUS (27S), self-association is significantly damaged [36]. Targeting the regulatory role and function of FUS phosphorylation in physiological and pathological LLPS could lead to the development of new therapeutic strategies.

Another notable point is that many studies have focused on phosphorylation in regulating protein interactions. In addition to relying on charge interactions, phosphorylation can also provide specific binding pockets for binding proteins. For example, in the PSD-95/SNAPAP/Shank complex, phosphorylation of the SAPAP N-terminal repeat mediates the PSD-95/SAPAP interaction, while multivalent interactions formed by postsynaptic density (PSD) scaffolding proteins lead to recombination PSD-like assemblies through phase separation, thereby promoting the formation of actin bundles [89, 90]. As another example, both Grb2 family proteins and PLCr1 contain an SH2 domain that can bind to phosphorylated LAT lysine residues to form a multi-protein complex, thereby regulating downstream signaling pathways [91]. From the initial stages of research on phase separation, several speculations about the influence of protein interaction on the phase separation were proposed [45]. Based on the polymer’s character, it is considered that multivalency drives phase separation. In this regard, the interaction between the PRM and SH3 is a good example, where the covalent and non-covalent interactions between PRM and SH3 influence polymer reassembly [23]. The intensity of phase separation was determined by the number of phosphorylated nephrin motifs and SH2/3 domains in the mixture. However, the principle of atomic interaction has not yet been explained. Subsequent studies stated that the proline-rich domain in SH3 supports the hydrophobic driving force [92]. In summary, phosphorylation regulates interactions by altering the positive charge, π bond stacking and provides binding pocket for partner proteins, then further affects LLPS. In addition to phosphorylation, acetylation has been reported to occur in multivalence-induced phase separation.

### Regulation of LLPS by acetylation of lysine and N-terminal

Another well-reported PTM in LLPS is acetylation, which regulates phase separation by neutralizing originally charged amino acids. Significantly, these proteins could be either positively or negatively charged because positively charged amino acids, such as serine, can be modified at the N-terminal [93]. Although acetylation on lysine alone shows many similarities with phosphorylation as they are both rapid and reversible, have no limitation on substrate, and are executed by a series of enzymes that are abundant in the cells, the difference is that the phosphate group confers amino acids with a negative charge, while the acetylation modification neutralizes the charged amino acid [94], and this difference accounts for the inhibitory effect of acetylation on phase separation. Another important point is that acetylation, similar to phosphorylation, affects phase separation by regulating the interaction between biological macromolecules; however, the underlying mechanisms are distinct.

**Fig. 6:**
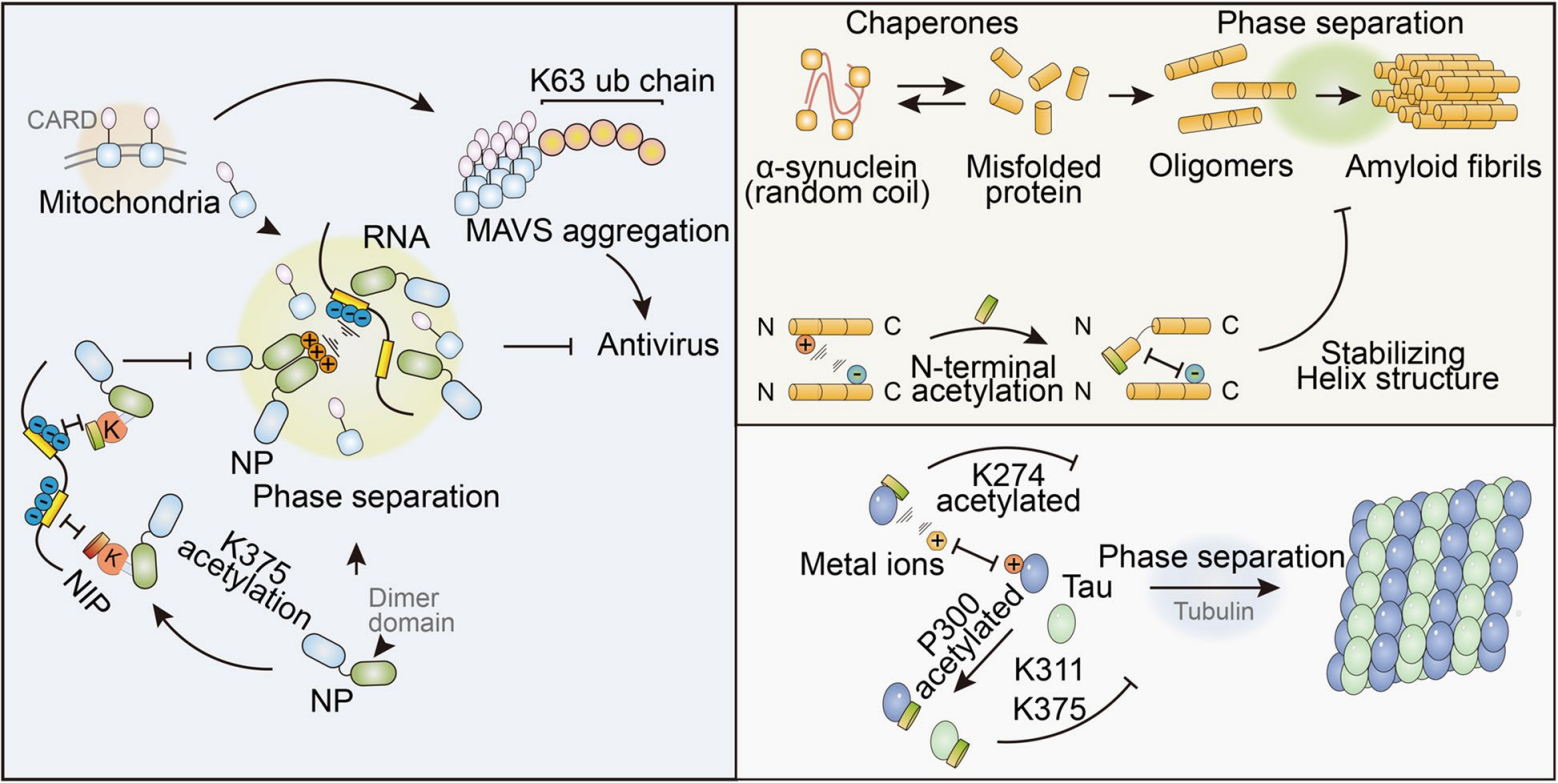
Acetylation in SYN and Tau phase separation. Naive SYN is misfolded and aggregated into oligomers, with the help of molecular chaperones. LLPS is crucial for the formation of amyloid fibrils formation and subsequent neurodegenerative diseases. N-terminal acetylation inhibits SYN from LLPS by neutralizing the positively charged N-terminal and stabilizing the Helix structure. Acetylation on K274 and K311/375 in Tau is also found to be inhibitory for its LLPS, either by enhancing ion binding affinity or hindering homo-interaction

α-Syn has long been discovered as a regulator of neurodegenerative diseases, including amyotrophic lateral sclerosis, Parkinson’s disease, and Alzheimer’s disease [95, 96]. Under pathological conditions, SYN proteins spontaneously interact with each other and form amyloid fibrils facilitated by phase separation [46, 97]. N-terminal acetylation inhibits Syn’s phase separation by either disrupting the interactions by stabilizing the helix structure or blocking long-distance N-C interactions. Therefore, acetylation may play a role in regulating electrostatic interactions. Competitive combinations also participate in phase-separation control. Another neurological disorder concerning the protein, Tau, is reported to be acetylated on K274, which facilitates metal binding with Zn^2+,^ Al^3+^, Cu^2+^, and Fe^3+^ ions [98]. Meanwhile the authors also discovered that KQ-mutated Tau tends to undergo phase separation [99, 100], as reported in the study of P62 phase separation. P62 is an autophagy receptor that undergoes phase separation via dimerization through the UBA domain. The acetylation of K420 and K435 enhances their binding affinity with ubiquitin and therefore blocks the interaction between UBAs (Fig. 6) [101].

Notably, since glutamine has similar amino acid side chains to lysine, most gain-of-function assays in these reports are simulated by the KQ mutation. These acetyl mimetics cannot accurately phenocopy lysine. They tend to be either over- or less functional. The distribution of electrons differs between acetylated lysine and glutamine, as glutamine is an ancestral residue for lysine in evolution [102].

**Fig. 7:**
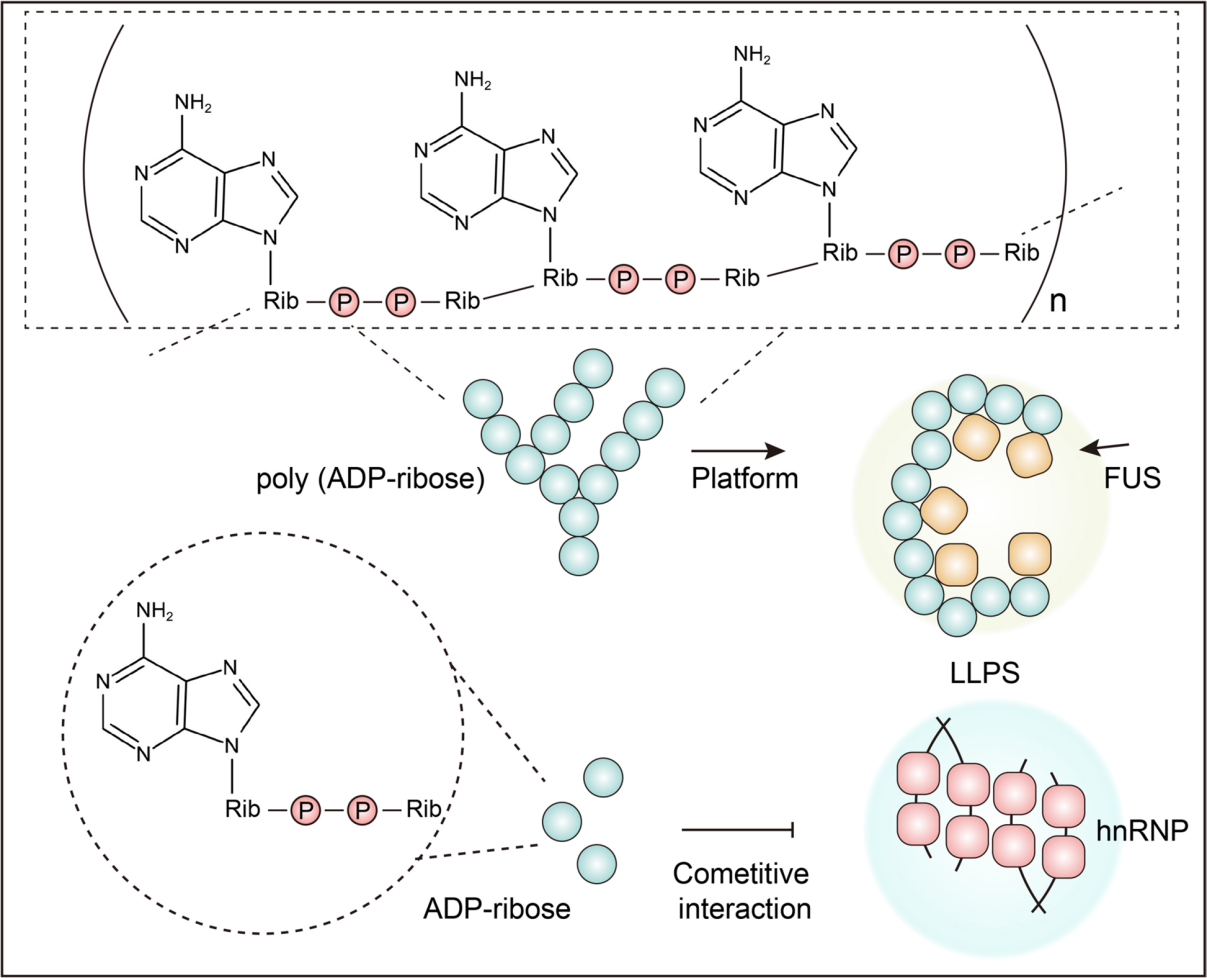
Poly ADP-ribosylation promotes LLPS. ADP-ribose is able to interact with or modify on substrates. Mono ADP-ribose modification on PAR-binding motif impedes hnRNP LLPS by competitive interaction. On the contrary, poly (ADP-ribose) chain provides an ideal platform for multivalent interaction and promotes the LLPS of FUS

Acetylation-regulated LLPS has also been found in SARS-CoV2019 infection. After invading host cells, NPs dispersed in the cytoplasm interact with each other and RNA through the dimer domain, thereby forming LLPS. These droplets suppress the host immune response by engulfing and inactivating MAVS. Its LLPS is controlled by acetylation because the uncharged acetyl groups can neutralize the originally charged lysine 375 and impede NP-nucleoid interactions. The CREB-binding protein (CBP) is considered to acetylate NPs. Unlike the KQ mutations employed in the aforementioned studies, the utilization of unnatural amino acid systems defines the effects of acetylation. Furthermore, the retro-inverso D peptide was designed to block K375 and mimic acetylation-induced inactivation (Fig. 7) [32].

For the in-depth understanding of acetylation in the regulation of phase separation, we considered Tau as an example. The first study on Tau acetylation and phase separation was executed in vitro in 2018, finding its inhibitory effect in impeding microtubule assembly [99]. Acetylation on K311/375, which lies on the amyloid core domain, catalyzed by P300 inhibits Tau aggregation and phase separation [100]. Although it inhibits over-aggregation, the authors speculate that the loss of function in microtubule assembly predominantly causes pathology. The following year, another study on lysine’s important function in phase separation and stress granule formation reached the same conclusion. The authors found a reversible function of acetylation in Tau, which decreases the interaction with RNA. Another study on Tau acetylation in 2019 drew a different conclusion, stating that the acetylation of K274 (KQ mutation mimic) enhances aggregation and filament formation while blocking tubulin interaction. In a follow-up study, changes in the K274Q mutation was further investigated to enforce its binding ability to metal ions, thereby impeding DNA protection [98].

The acetylation of the N-terminal merits further discussion. The acetylation of lysine is highly conversed from prokaryotes to eukaryotes. In contrast, N-terminal acetylation is more unique in prokaryotes. N-Acetylation is found in 85% of mammalian proteins and 60% of yeast proteins, making it one of the most abundant protein modifications in the cells. In-vitro experiments predominantly using truncated or primary nuclear purified proteins are often employed for phase separation studies, which makes N-acetylation likely to be artificially ignored [103]. Moreover, owing to the solution charge, purifying stable proteins through N-acetylation remains technically difficult [104]. In the study of FUS, a nuclear magnetic resonance spectroscopy assay shows that N-acetylation does not change the FUS disorder structure but sightly promotes phase separation. But the aggregation of FUS is intensively suppressed and therefore damages FUS liquid-gel transition [103]. The N-terminal is a key factor in changing its N-terminal charge and aggregation and therefore influences its LLPS.

### Positive regulation of LLPS by poly (ADP-ribosyl)ation

Poly (ADP-ribosyl)ation is a reversible and highly conserved PTM in eukaryotes, prokaryotes, and even viruses [105]. It takes part in a series of biological processes, including immune responses as well as inflammation and DNA damage repairing with the help of ADP-ribosyltransferase (ART) superfamily [105]. It has the potential of controlling LLPS owing to the strong neutralizing effect of charged amino acids. ADP-ribose (ADPr) from NAD precursors can be covalently bound to the side chains of arginine, serine, and aspartic acid through ester linkages [106]. New ADPr iteratively connect to the ADPr units that are already modified on the substrate, forming a long chain of up to 200 repeats [107]. For molecules that can bind to ADPr, the long ADPr chain offers an ideal multivalent platform for LLPS [108, 109]. In the study of apoptosis signal-regulating kinase 3 (ASK3), PAR was shown to cause an osmotic stress through promoting LLPS to maintain the cell volume. When cells are subjected to a hyperosmotic stress, ASK3 undergoes LLPS and achieve inactivation, which prove that PAR is crucial for maintaining the dynamics. Mechanistically, ASK3 interacts with PAR through two arginine residues on the PAR-binding motif (PBM), which weakens the interaction with itself [110]. Another study on FUS found that FUS is attracted to DNA lesions after arginine/glycine-rich domains interact with PAR chains. In addition, the RGG2 domain is important for ADPr interaction and FUS LLPS in DNA repair [111].

### The opposite regulation of ubiquitination and methylation for LLPS

The ubiquitination and methylation are also found to regulate LLPS [39, 112–116]. The multivalent binding potential of the ubiquitin molecule and the characteristics of long molecular chains make its LLPS unique [57]. In the emergency response of eukaryotic cells, molecules associated with protein degradation are often observed to rapidly coalesce together, forming stress granules (SGs). By wrapping the corresponding proteins and nucleic acids, the normal physiological activities of the cells are suspended, and a protective effect is exerted. Ubiquitination is a form of regulation for the urgent response [117]. In the study of substrate-shuttling factor RAD23B, ubiquitination is found to be crucial in the formation of proteasome and LLPS [53]. In contrast to other functions of PTMs in changing the electronic characteristics or aggregation form of substrate, the ubiquitin chain itself is considered to be essential in regulating proteolysis.

Methylation is a broad and reversible PTM that is commonly found on the carboxyl groups of glutamate and leucine or on the guanidinium ion of arginine, histidine, and lysine [39, 54]. Methylation can strongly neutralize the original electrical properties of proteins and hinder electrostatic action. Therefore, it strongly inhibits the LLPS of the substrate. In the study of RNA-binding proteins, the cation–π interactions of RGG/RG-rich motifs were found to be important in regulating phase separation, and asymmetric di-methylation on arginine can weaken the driving force and phase separation of RBP, thereby increasing the granule dynamics of RNP [24, 118].

## The potential of LLPS in treating neurodegenerative diseases and cancer

**Fig. 8:**
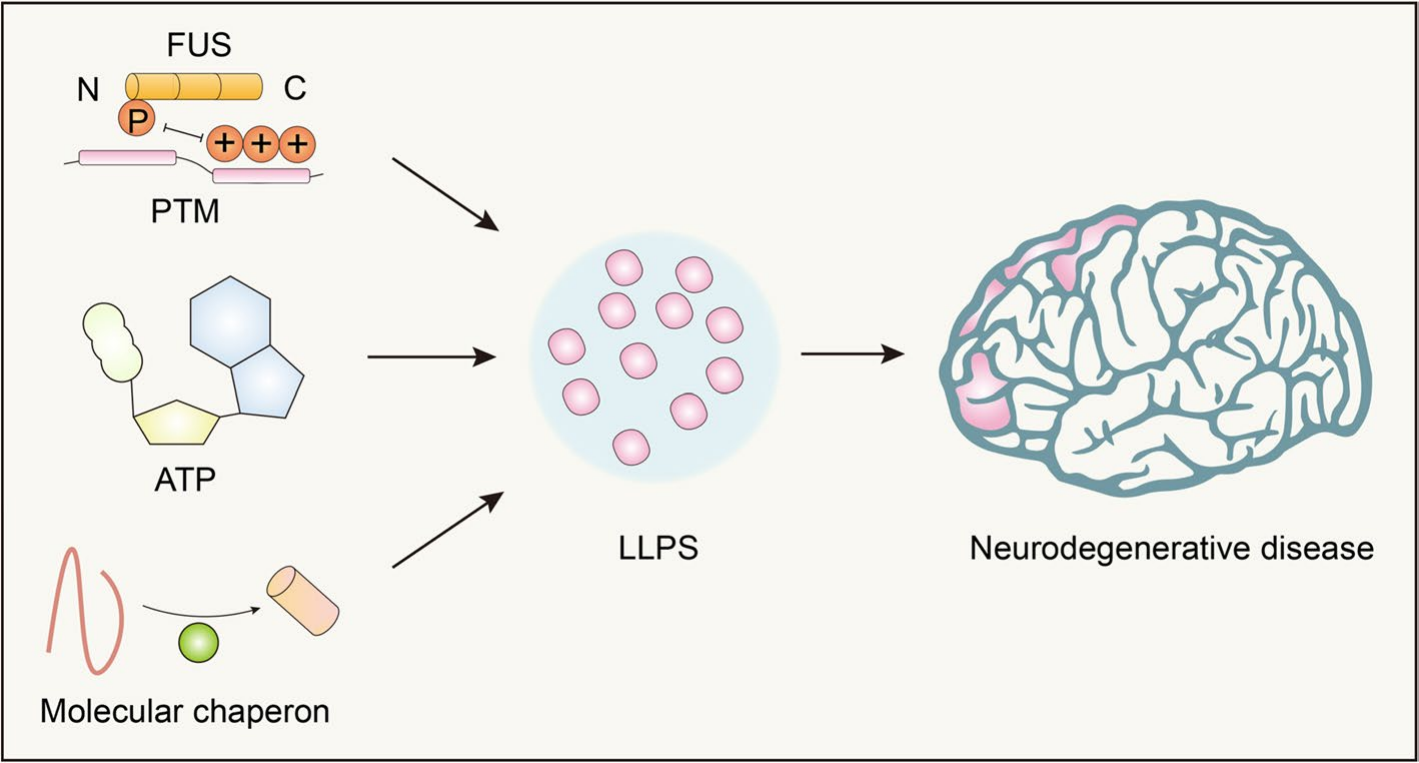
Factors in regulating LLPS-induced neurodegenerative diseases. Neurodegenerative disease such as amyotrophic lateral sclerosis, Parkinson’s disease, and Alzheimer’s disease are reported to be controlled by LLPS. Different circumstances are important in regulating this process. The PTM, specifically speaking, the phosphorylation of FUS enhances its LLPS. Chaperon such as HSP70 promotes the misfolding and subsequent LLPS of α-synuclein. Aging leads to a decrease in ATP levels, which reduces the reversibility of cellular LLPS

Biopolymer condensates can be converted into various material states. This is important for realizing unique functions, such as biochemical reaction centers, signaling centers, and supporting structures [119]. Abnormal phase separation and transition are related to various human diseases, including neurodegenerative diseases (Fig. 8) and cancer [117]. Protein aggregation is a characteristic of neurodegenerative diseases. However, the mechanisms underlying this pathogenic aggregation remain unclear. In recent years, neurodegenerative diseases have been found to aggregate pathological proteins with the ability to facilitate LLPS under physiological conditions. However, these proteins are likely to cause toxicity because of changes in their behavior in neurodegenerative disease-related variants and states [120]. Therefore, therapeutic approaches to combat abnormal LLPS may alleviate the toxicity and aggregation prevalent in neurodegenerative diseases. There are several ways to treat neurodegenerative diseases by altering the LLPS.

Molecular chaperones, such as heat shock proteins and nuclear proteins, are involved in the protein folding, protein degradation, translation of nascent peptides, and return of misfolded proteins to their original form. Proteases help to maintain stability through a variety of mechanisms [121]. In some special cases, molecular chaperones may possess a protein depolymerase activity that can restore natively folded proteins from aggregated states. Furthermore, harnessing the power of molecular chaperones may be a promising therapeutic strategy, as neurodegenerative diseases are associated with an inability to maintain a protein balance [122, 123]. On the one hand, researchers have reversed the pathological aggregation of neurodegenerative disease-related proteins, such as TDP-43, FUS, and α-synuclein, through genetic engineering methods, such as enhancing the activity of Hsp104 depolymerase [98, 124]. Another recently discovered human protein-depolymerase system, with a three-part motif (TRIM) protein composition, enhanced TRIM11 depolymerase activity in conjunction with sumo ligase activity to degrade abnormal proteins and existing deposition, including amyloid fibrils [125]. On the other hand, inducible chaperone expression is stimulated by small-molecule drugs that penetrate the brain to induce the transcription of specific chaperones [126]. Several drugs that enhance chaperone expression have been developed, and some of them are in clinical trials for the treatment of ALS, including arimoclomal, a co-inducer of Hsp70 and Hsp90, and colchicine, which generally induces HspB8 expression, thereby slowing the deterioration of ALS [127, 128].

Recent studies have shown that nuclear protein abnormalities are associated with the mislocalization, and accumulation of proteins related to neurodegenerative diseases. In addition to the classical functions of nuclear import and export, nuclear proteins act as chaperones to prevent misfolding, accumulation, and irreversible LLPS. For example, nucleoprotein-β2 (Kapβ2) can prevent and reverse FUS, hnRNPA1, hnRNPA2, TAF15, EWSR1, etc. phase separation [118, 129, 130]. In terms of ATP levels, aging leads to a decrease in ATP levels, which reduces the reversibility of cellular LLPS by reducing the dynamics of stress granules [131, 132]. This loss of reversibility significantly increases the risk of developing neurodegenerative diseases. However, increasing intracellular ATP levels, such as chemically hydrophilicity, can directly inhibit phase separation and protein aggregation, thereby combating neurodegenerative diseases [133].

There are several cases of neurodegenerative disease-related proteins, whose LLPS levels and aggregation state may be altered by PTMs. Charge changes induced by complementing the phosphorylation of proteins facilitate LLPS driven by charge-charge interactions [42]. This may also hinder LLPS because of charge repulsion and steric hindrance caused by different positions of the phosphate group [63]. The phosphorylation of serine/threonine residues in TIA1 and Tau promotes LLPS [70, 134]. Conversely, the phosphorylation of the prion-like domain (PrLD) of FUS reduces LLPS [135]. Specifically, the phosphorylation of FUS PrLD introduces static charges that inhibit multivalent interactions driving LLPS, which is a process that reduces the risk of neurodegenerative disease [41, 136, 137]. Similarly, the phosphorylation of TDP-43 PrLD inhibits the number of aggregates in cells [138], and its phase separation is also regulated by PTMs in the N-terminal domain, where the phosphorylation of Ser48 at the N-terminus inhibits LLPS [37]. In addition, poly (ADP-ribosyl)ation is an important PTM that regulates stress particle dynamics. The inhibition of poly (ADP-ribose) polymerase can reduce the phase separation level of cytoplasmic TDP-43 and related neurotoxicity, thereby reducing ALS in NSC-34 cells, rat primary spinal cord neurons, and fruit fly models [124, 139, 140]. Changes in methylation sites can also regulate LLPS and the toxicity of FUS or DPRs [118, 141, 142]; for example, PRMT reduced FUS LLPS levels in vitro by adding methyl groups to arginine residues [118, 143]. Notably, specific concentrations of global methyltransferase inhibitors can reduce the aggregation of FUS mutants in cells [144]. In summary, these findings suggest that specific PTM upregulation or downregulation through genetic or pharmacological interventions may be an attractive strategy for the treatment of abnormal LLPS. Therefore, PTMs of ALS-related proteins should be explored further to identify potential therapeutic targets.

LLPS in the field of cancer research, especially in therapeutic strategies, has rarely been studied. Chemotherapy is an effective treatment for cancer, but the mechanisms by which drug resistance develops are not fully understood. Recent studies have shown that the selective separation of chemicals or the concentration of therapeutic drugs in condensate may affect the drug concentration and activity. By altering the properties of the condensate, the activity of the drug can be modulated, which could aid research on cancer treatment and drug resistance. For example, in ER+ breast cancer, the physicochemical properties of tamoxifen, which is independent of the agent target, facilitate its accumulation in MED1 condensates, preventing Erα incorporation into the condensates, and to a certain extent, induce MED1 condensate exclusion to inhibit cancer progression. However, the large condensates formed by the overexpression of MED1 diluted the concentration of tamoxifen, leading to the development of drug resistance. Conversely, the deregulation of MED1 increases drug concentrations in aggregates, restoring chemosensitivity to tamoxifen in breast cancer cells [145].

## Conclusions and perspectives

In the past decade, research on LLPS has led to many unexpected and fruitful outcomes. Advancements from the initial observation of micron-level droplets to the organelle-like hypothesis to the later intermolecular interaction forces, our understanding of LLPS has gradually moved from macro to microlevels. However, as understanding deepened, more questions emerged. For example, how do different stimuli coordinate to regulate LLPS? Will there be an impact between the LLPS of different proteins? How can the study of LLPS be applied to address this disease?

Research on the relationship between PTMs and LLPS is essential. Although the regulatory role of PTMs in LLPS has been reported, comprehensive research remains limited, and more LLPS-related PTMs need to be investigated. Based on evolutionary relevance and existing literature reports, we summarized the theoretical rationale for the relationship between PTMs and LLPS and aimed to provide a systemic idea for future researchers. Thus far, PTMs, including phosphorylation, ubiquitination, acetylation, and methylation, have been reported to regulate LLPS; however, based on the evolutionary relationship between PTM and LLPS described above, we believe that several PTM types are yet to be discovered. Several reviews have made similar assumptions about this conjecture on different theoretical bases, particularly for membrane proteins owing to their two-dimensional distribution, easy aggregation, and wide range of modifications. The reason for the delay in reporting may be because of the limitations of the detection methods or technical conditions. O-glycosylation is a common PTM that links glycosyl with the hydroxyl groups on the amino acid side chain [146]. The hydroxyl groups that can be used for bonding are mainly alcohol hydroxyl groups of serine and threonine, hydroxyl groups of hydroxyl lysine, and phenolic hydroxyl groups of tyrosine. N-glycosylation is pre-synthesized and then transferred to the acceptor peptide as a whole, but O-glycosylation is usually transferred to the corresponding residues one by one [147]. The process of joining glycosyl molecules is complicated, as all the 10 monosaccharides can be modified in a linear sequence. Both elongated glycosylation chains and amino sites on the substrate provide platforms for multivalent binding. However, the strong electronic neutralization effect of O-glycosylation may block potential LLPS.

Research on LLPS is still in its infancy stage, and the regulation of LLPS by PTM will undoubtedly be a focus of future research. Drugs for PTM have benefited many patients, and it is expected that PTM-related research will facilitate the clinical applications of LLPS in near future.

## Data Availability

Not applicable.

## Abbreviations

LLPS: Liquid-liquid phase separation
PTMs: Post-translational modifications
STINGs: Stimulator interferon genes
IDRs: Intrinsically disordered regions
PRM: Proline rich motif
SH3: Src homology 3
ROS: Reactive oxygen species
α-Syn: α-synuclein
PAR: Poly (ADP-ribosyl)ation
FUS: Fused in sarcoma
H1: Histone H1
FMRP: Fragile-X mental retardation protein
CARPN1: Calcium-activated neutral proteinase 1
LAT: Activation of T cells
Grb2: Growth-factor-receptor-bound protein 2
SOS: Sevenless
hnRNPA2: Heterogeneous nuclear ribonucleoprotein A2
PSD: Postsynaptic density
CBP: CREB-binding protein
ART: ADP-ribosyltransferase
ADPr: ADP-ribose
ASK3: Apoptosis signal-regulating kinase 3
PBM: PAR-binding motif
SGs: Stress granules
TRIM: Three-part motif
Kapβ2: Nucleoprotein-β2
PrLD: Prion-like domain
